# Structural Dynamics Descriptors for Metal Halide Perovskites

**DOI:** 10.1021/acs.jpcc.3c03377

**Published:** 2023-08-30

**Authors:** Xia Liang, Johan Klarbring, William J. Baldwin, Zhenzhu Li, Gábor Csányi, Aron Walsh

**Affiliations:** † Department of Materials, Imperial College London, South Kensington Campus, London SW7 2AZ, U.K.; ‡ Department of Physics, Chemistry and Biology (IFM), Linköping University, Linköping SE-581 83, Sweden; § Department of Engineering, University of Cambridge, Cambridge CB2 1PZ, U.K.; ∥ Department of Physics, Ewha Womans University, Seoul 03760, Korea

## Abstract

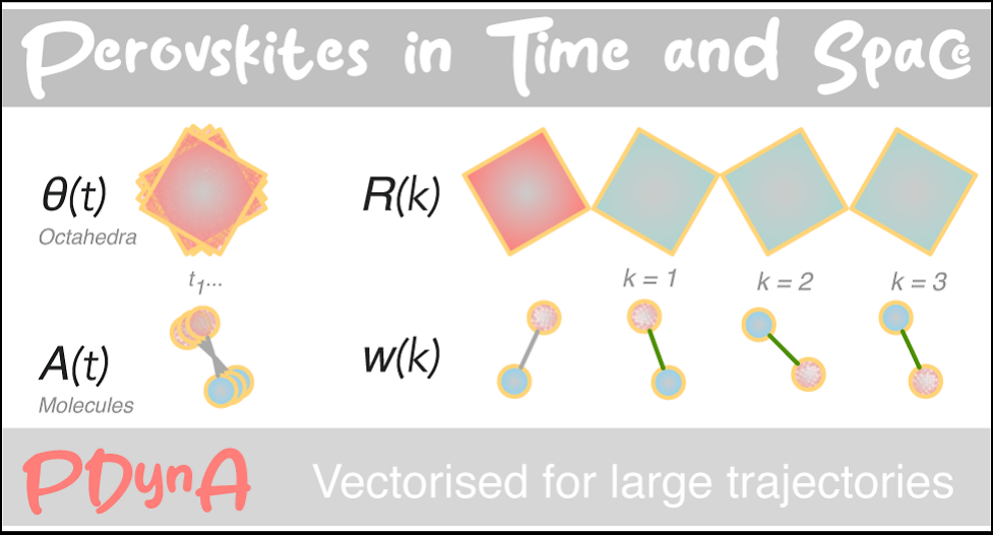

Metal halide perovskites have shown extraordinary performance in solar energy conversion technologies. They have been classified as “soft semiconductors” due to their flexible corner-sharing octahedral networks and polymorphous nature. Understanding the local and average structures continues to be challenging for both modeling and experiments. Here, we report the quantitative analysis of structural dynamics in time and space from molecular dynamics simulations of perovskite crystals. The compact descriptors provided cover a wide variety of structural properties, including octahedral tilting and distortion, local lattice parameters, molecular orientations, as well as their spatial correlation. To validate our methods, we have trained a machine learning force field (MLFF) for methylammonium lead bromide (CH_3_NH_3_PbBr_3_) using an on-the-fly training approach with Gaussian process regression. The known stable phases are reproduced, and we find an additional symmetry-breaking effect in the cubic and tetragonal phases close to the phase-transition temperature. To test the implementation for large trajectories, we also apply it to 69,120 atom simulations for CsPbI_3_ based on an MLFF developed using the atomic cluster expansion formalism. The structural dynamics descriptors and Python toolkit are general to perovskites and readily transferable to more complex compositions.

## 1 Introduction

Perovskite is one of the most important classes of crystal structures in materials chemistry owing to the large number of accessible compositions and variety of structural derivatives. For standard ABX_3_ compounds, the A-site cation is positioned inside a cuboctahedral cage formed of corner-sharing BX_6_ octahedra. When the X-site is occupied by a halide, the A cation is monovalent, and the B cation is divalent, the material is called a metal halide perovskite. The term hybrid organic–inorganic halide perovskite can refer to compounds where the A-site is occupied by a molecular cation, *e.g.*, formamidinium (FA^+^) or methylammonium (MA^+^), while the B-site cation is an inorganic cation such as Pb^2+^ or Sn^2+^. These perovskites have become a popular research topic in the context of photovoltaics and related optoelectronic technologies.^1^

The cubic perovskite archetype structure (space group, *Pm*3*m*), which features a corner-sharing array of regular octahedra, is typically only observed at higher temperatures. A series of lower-symmetry perovskite phases emerge at lower temperatures. These polymorphs can be classified according to the average octahedral tilting patterns^2−4^ and the types of ion displacements that occur.^5^ The instantaneous structure often differs from the structure averaged over space and time, which can be seen from differences between X-ray diffraction measurements and more local probes such as electron diffraction,^6^ X-ray scattering,^7^ Raman spectroscopy,^8^ and nuclear magnetic resonance.^9^

There are advantages to using hybrid perovskites in photovoltaic devices, such as long photogenerated carrier diffusion lengths, which have been related to the polar nature of the organic A-site cations.^10,11^ However, the presence of molecular species introduces an additional degree of rotational freedom in the crystal. The structure of light elements (*e.g.*, C, H, and N) can be difficult to distinguish from the heavy frameworks (*e.g.*, Pb, Br, and I) using standard characterization techniques such as X-ray diffraction. The molecular arrangement and reorientations have been probed from techniques including quasi-elastic neutron scattering^12^ and time-resolved infrared spectroscopy.^13^ Compositional engineering of perovskites to produce stable and high-performance materials has led to the study of complex mixtures such as Cs_0.05_FA_0.78_MA_0.17_Pb(I_0.83_Br_0.17_)_3_.^14^ Experimental observations on these state-of-the-art materials suggest that the (average) cubic perovskite phase of this material possesses (local) symmetry-breaking tilting of octahedra that hinders degradation pathways.^15^

From the materials simulation perspective, modeling of perovskite crystal dynamics at finite temperatures can be achieved using molecular dynamics (MD).^16−21^ However, large and long simulations are required to quantify local fluctuations in atomic positions and correlations, for example, between rotations of the molecular sublattice and tilting of the octahedral inorganic networks. MD simulations based on density functional theory (DFT) forces are restricted in system size (typically 10s to 100s of atoms for 100s ps) due to the high computational cost, and conventional force fields are lacking in their ability to describe both the dynamics of the organic A-site cations and the highly deformable B-site octahedra. Alternative methods are necessary to enable the collection of accurate and efficient structural information.

### 1.1 Machine Learning Force Fields

Traditional empirical descriptions of interatomic interactions rely on a fixed functional form, such as Lennard-Jones or Buckingham potentials.^22^ Machine learning force fields (MLFFs) involve a more flexible model to learn and predict quantities such as energies, atomic forces, and stresses (EFS) of crystal structures from an input (training) dataset.^23^ MLFFs are promising to reach a DFT-level accuracy with an efficiency comparable to classical potentials. In such approaches, the atomic structures are converted into a symmetrized feature space, and functions on these spaces thus can be learned with general ML regression algorithms.

Gaussian process regression is a powerful tool for non-linear function approximation in high-dimensional parameter spaces.^24^ It is used in the Gaussian approximation potential (GAP) formalism, where the local environments of atoms in a crystal structure are converted into a structural feature space.^25,26^ It is commonly used in combination with a local atom-centered representation called smooth overlap of atomic positions (SOAP),^27^ which incorporates two-body radial and three-body angular contributions. The reference configurations for force field training can be selected in several ways, including through the use of Bayesian error estimation. Based on this approach, Jinnouchi *et al.*(28) implemented an on-the-fly MLFF method with DFT-level accuracy in predicting the energy and forces of atomic configurations and is *ca.* 100 times faster than standard DFT calculations. There have been prior successes in the application of this approach to metal halide perovskites.^29^

### 1.2 Perovskite Structural Descriptors

The temperature dependence of the unit cell parameters is a commonly tracked feature of crystallographic perovskite phases. However, a quantitative description of the underlying octahedral deformations and the A-site cation dynamics is required to reveal more important details connected to materials properties and performance.

The common language for classifying octahedral tilting is the Glazer notation,^2^ where three-dimensional tilting can be described with three magnitudes, each denoting the tilting about *a*, *b*, and *c* axes, and the corresponding in-phase(+)/anti-phase(−) tilting correlation pattern along that direction. For instance, the Glazer notation of an orthorhombic perovskite phase can be *a*^+^*b*^–^*b*^–^, meaning that the tilt angles about *b* and *c* axes are the same and different from that of *a* axis, and the tilting about *a* axis is in-phase and the other two directions are anti-phase. Octahedral tilting can also be described using the B–X–B bond angle^30^ or the X–B–B–X dihedral angle.^31^

Beyond the analysis of static or globally averaged structures (coordinates averaged in time and space), the temporal and spatial behavior of perovskite structures are of growing importance for understanding inhomogeneities in perovskite materials and performance bottlenecks in perovskite devices.^32^

Here, we developed the perovskite dynamics analysis (PDynA) software package to systematically quantify the dynamic behavior of perovskite materials in an automatic and efficient manner. The integrated analysis tools aim to create an intuitive picture of the structural dynamics, as well as to provide compact descriptors for future data-driven studies of perovskites. Such analysis can provide validation for MLFFs and systematically interpret MD trajectories. The package combines standard quantification methods with novel descriptors to build a full view of the structural dynamics in perovskite crystals.

## 2 Methods

### 2.1 Perovskite Dynamics Analysis

PDynA is an integrated Python code that is tested with Python 3.8+. The algorithm first identifies all constituent octahedra and their connectivity as well as the A-site cations in the perovskite structure. We then formulate a set of compact descriptors to represent the structural characteristics in space and time, which can be compared for different external conditions and chemical compositions.

Functions are developed to analyze MD trajectories in four general aspects: lattice parameters (relative position of BX_6_ octahedra), octahedral tilting, octahedral distortion, and A-site molecular orientation and displacement. The computation of these properties is vectorized with multi-threading to enable faster processing of large trajectories. The analysis of dynamic structural behavior can be done in two modes, static (time-independent) analysis mode and transient (time-dependent or temperature-dependent) analysis mode. In the static mode, the behavior of constant-temperature MD trajectory will be sampled after a given equilibration period and outputs static properties in the form of cumulative data. The sampling frequency is automatically determined according to the system size. In the transient mode, computed properties will be sampled evenly across the trajectory and provide structural information with respect to time or temperature.

In this work, *a*, *b*, and *c* are the principal axes related to crystallographic perovskite phases. These three axes have a one-to-one correspondence to the principal Cartesian axes of the sample, *x*, *y*, and *z* unless specified otherwise. Under most circumstances, these two systems have one-to-one correspondence except during a phase-transition event (*e.g.*, when cooling from a cubic to a tetragonal phase, a tetragonal distortion may emerge along *x*, *y*, or *z* of the simulation cell). We use α and β to denote variable symbols associated with one of the sample-specific axes.

#### 2.1.1 Lattice Parameters

**Figure 1:**
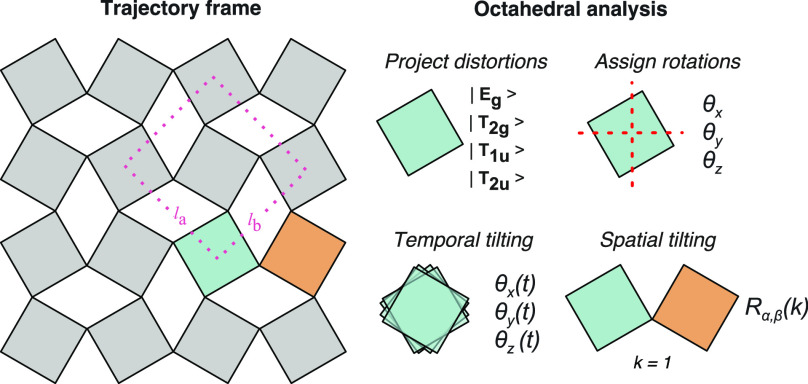
Illustration of the procedure for structural information extraction from a MD trajectory frame of an ABX_3_ perovskite, including the definition of local lattice spacing from the relative positions of the B atoms and the assignment of BX_6_ octahedral tilting.

Lattice parameters of the unit cell are straightforward indicators of crystallographic phases for perovskite materials. These can be defined globally from the dimensions of the supercell or locally from the atomic positions in the MD trajectory. We calculate three local lattice spacings *l*_a_, *l*_b_, and *l*_c_ from the relative pairwise positions of the B-site cations following ref (33) and illustrated in Figure 1. These local spacings can be converted into three (pseudo-cubic) local lattice parameters *l*_pc,a_, *l*_pc,b_ and *l*_pc,c_ with the relationships .

#### 2.1.2 Octahedral Distortions

An obstacle to the analysis of perovskite structures is that the BX_6_ octahedral building blocks are not rigid but deform in time. Group theory can be applied to construct a quantitative and compact measure of octahedral distortions. Magnitudes of the four irreducible distortion modes, namely *E*_g_, *T*_2g_, *T*_1u_, and *T*_2u_, are computed for each octahedron in space and time, following the procedure outlined by Morita *et al*.^34^ The function uses the Hungarian order matcher method^35^ as implemented in pymatgen.^36^ Gaussian peaks are fitted to each population to determine the mean and standard deviation of the dynamic distortion behavior of the structure. Also, the octahedral distortions of the *time-averaged* structure can be calculated to analyze static global distortions in the phase of interest.

#### 2.1.3 Octahedral Tilting

Together with the identification and removal of distortion modes, each octahedron is matched to an ideal reference configuration. We created a function to simultaneously analyze the octahedral tilting and distortions and output the three-dimensional tilting status as a rotation matrix. This method follows the resolved connectivity of B- and X-sites and avoids anomalies arising from the structural mismatch, differing from the implementation used in ref (34).

We denote the individual tilting angle of an octahedron around axis α at time *t* as
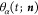
1where ***n*** = (*n*_*x*_, *n*_*y*_, *n*_*z*_) are 3 integer coordinates indexing the octahedra in the supercell. We identify the tilting angles in eq 1 with the three Euler rotation angles computed from the rotation matrix. By symmetry, θ_α_(*t*; ***n***) takes the range −45° < θ_α_ < 45°. The tilting of octahedra is spatially correlated between neighbors, a crucial feature in the understanding of perovskite phases. We track the tilting angle relationship between an octahedron ***n*** and its *k*th neighbor in the *x* direction using the expression
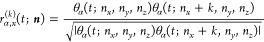
2and similarly for tilts in the *y* and *z*-directions. We record the distribution of *r*_α,*x*_^(*k*)^(*t*; ***n***) over all octahedra, all timesteps, and over ±*k* along an MD trajectory. This distribution can then be plotted as a function of the tilt angle. The sign of *r*_α,β_^(*k*)^, β = *x*, *y*, or *z*, reflects the nature of in-phase (positive) or anti-phase (negative) correlation. For the special cases of α = β and *k* = 1, the correlation reflects the first nearest neighbor tilt angle along the tilt direction, which is related to the superscript of the Glazer notation. For α ≠ β, *r*_α,β_^(*k*)^ reflects the off-axis correlation, *i.e.*, the relationship between tilts of neighboring octahedra in a plane orthogonal to the tilting direction.

We further calculate global spatial tilting correlation functions as
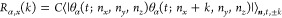
3where ⟨...⟩_***n***,*t*,±*k*_ denotes an average over all octahedra in the supercell over simulation time and over neighbors in both the positive and negative *x* directions and *C* is a constant such that *R*_α,β_(0) = 1. The spatial extent of the correlation of octahedral tilts can be quantified by fitting *R*_α,β_(*k*) to a decaying exponential
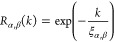
4where ξ_α,β_ is the fitted correlation length in the β direction of the tilt around the α-axis. ξ_α,β_ forms a second rank tensor with nine components.

We define the tilting correlation polarity (TCP) around direction α, δ_α_, by comparing the positive and negative correlation population counts from *r*_α,α_^(1)^ (eq 2)
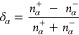
5where *n*_+_ and *n*_–_ are the counts of positively (*r*_α,α_^(1)^ > 0) and negatively (*r*_α,α_^(1)^ < 0) correlated octahedron pairs, respectively. As a result, δ_α_ ranges from −1 to 1. Values of δ_α_ close to 1, 0, and −1 correspond to Glazer notation superscripts of +, 0, and −, respectively.

#### 2.1.4 Molecular Orientation

For hybrid compositions, the molecular components can rotate in three-dimensional space. One or multiple vectors are used to describe the molecular orientation (MO). The molecules currently implemented are MA and FA, but an interface is provided for user-defined molecules. For MA (CH_3_NH_3_), a single vector ***v***_MA_ connects the C and N atoms and is sufficient to fully describe the rotation (if the contribution from hydrogen atoms is ignored). For FA (CH(NH_2_)_2_), two vectors are required to fully describe the MO. The polarization vector ***v***_FA1_ is the vertical bisector from the C atom to the connection between the two N atoms. The second vector ***v***_FA2_ connects the two N atoms.

We denote the unit length MO of a molecule at time *t*, ***v***(*t*; ***n***), where ***n*** = (*n*_*x*_, *n*_*y*_, *n*_*z*_), again, are 3 integer coordinates indexing the molecules in the supercell. Akin to the case of octahedral tilting, we track the spatial correlation of MOs between *k*th neighbors in the *x* direction at time *t*
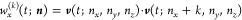
6and similarly for neighbors in the *y* and *z* directions. The distribution of *w*_α_^(*k*)^, α = *x*, *y*, or *z*, over all octahedra and simulation time is denoted *C*_α_^(*k*)^(*w*). Based on this distribution, we define two quantitative descriptors for the spatial correlation MOs: the alignment factor (AF) and the contrast factor (CF). The AF in the α direction is
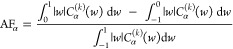
7and quantifies the relative difference between the tendency of aligned [∫_0_^1^|*w*|*C*_α_^*k*^(*w*)d*w*] and anti-aligned [∫_–1_^0^|*w*|*C*_α_^*k*^(*w*)d*w*] nearest-neighbor molecular order. The CF measures the similarity of the relative alignment of the first- and second-nearest neighbor in the direction α
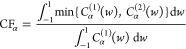
8

This quantity indicates the similarity between the first and second nearest neighbor distributions, *C*_α_^(1)^ and *C*_α_^(2)^, by taking the overlapping portion of them as a fraction of the entire population. We then average CF_α_ over the three Cartesian directions
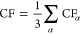
9

CF and AF take values between in the ranges [0, 1] and [−1, 1], respectively. We choose to rescale  to map it onto [−1, 1] to make plotting and comparison between the two descriptors more convenient.

We further compute the temporal autocorrelation of the molecular orientation
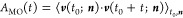
10

The characteristic times associated with the reorientation of the MOs are calculated by fitting this autocorrelation to a sum of two exponential decays:^33^
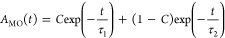
11where *C* is a fitted constant for tuning the linear combination of the two exponential functions and τ_1_ and τ_2_ are time constants. Typically, *C* is approximately 0.9 after parameter optimization, indicating that τ_1_ is the dominant time scale, while the second exponential term acts as a correction term for the fast initial decay, with typical values of τ_2_ around 0.5 ps. Therefore, the calculated time constant for the observed decaying autocorrelation function is the fitted parameter τ_1_.

In addition to these core functions, PDynA also contains functions that calculate the *time-averaged* structure, radial distribution function, A-site cation displacement, composition heterogeneity analysis, three-dimensional visualization of MO and tilt domain, *etc*. The visualization of atomic structures in this work is performed with VESTA,^37^ and Matplotlib^38^ is used for plotting the rest of the results.

### 2.2 MLFF Training and Trajectories for CH_3_NH_3_PbBr_3_

MD trajectories are generated for CH_3_NH_3_PbBr_3_ using a custom MLFF. The MLFF is based on Gaussian process regression with Bayesian error estimation^28^ as implemented within the Vienna *ab initio* simulation package (VASP).^39,40^ The EFS used for the training data are calculated based on DFT. Projector augmented wave (PAW) potentials are used for all DFT calculations. The chosen PAW potentials for the involved elements are Cs_sv (5s^2^5p^6^6s^1^), C (2s^2^2p^2^), N (2s^2^2p^3^), H (1s^1^), Pb_d (5d^10^6s^2^6p^2^), I (5s^2^5p^5^), and Br (4s^2^4p^5^), with all projection operator evaluations in reciprocal space. The r^2^SCAN exchange–correlation functional^41^ is selected. The energy threshold for electronic convergence is set to 10^–5^ eV, and the plane wave basis set cut-off energy is 500 eV. Gaussian smearing with a width of 50 meV is adopted for the smearing of electronic band occupancy. A 2 × 2 × 2 Γ-centered *k*-point grid is used for all training calculations.

For the MD simulations, an isothermal–isobaric (NpT) ensemble with an external pressure of 1 bar is first adopted to acquire equilibrium lattice parameters and atomic positions on a 2 × 2 × 2 supercell (96 atoms) at each temperature of interest, and the initial structure is collected from the Materials Project database.^42^ This MD calculation is accelerated with the on-the-fly learning MLFF, and the local configurations collected in this step are neglected. The equilibrium configuration (average lattice parameters and atomic positions at that temperature) is then imported into another isothermal–isochoric (*NVT*) ensemble with the same supercell size. *Ab initio* data for force field training is also retrieved from the *NVT* MD using the on-the-fly learning mode. A Langevin thermostat is applied to both ensembles, and atomic and lattice friction constants are set to 10 ps^–1^. The MD time step for hybrid halide perovskites is set to 0.5 fs, and 100,000 steps are performed in each MD calculation.

Independent on-the-fly MLFF training processes are performed for the constituent perovskite polymorphs at 100 K (γ), 160 K (β), 210 K (β), and 350 K (α). On-the-fly learning is achieved through Bayesian error estimation, where a DFT calculation will be performed only if the predicted error is above a threshold.^28^ The training data picked up from all four on-the-fly MD runs are combined into a final training set used to produce a general force field. The resulting training set contains 1520 DFT snapshots, from which 690, 602, 3729, 780, and 129 local reference configurations for Br, C, H, N, and Pb are extracted.

**Figure 2:**
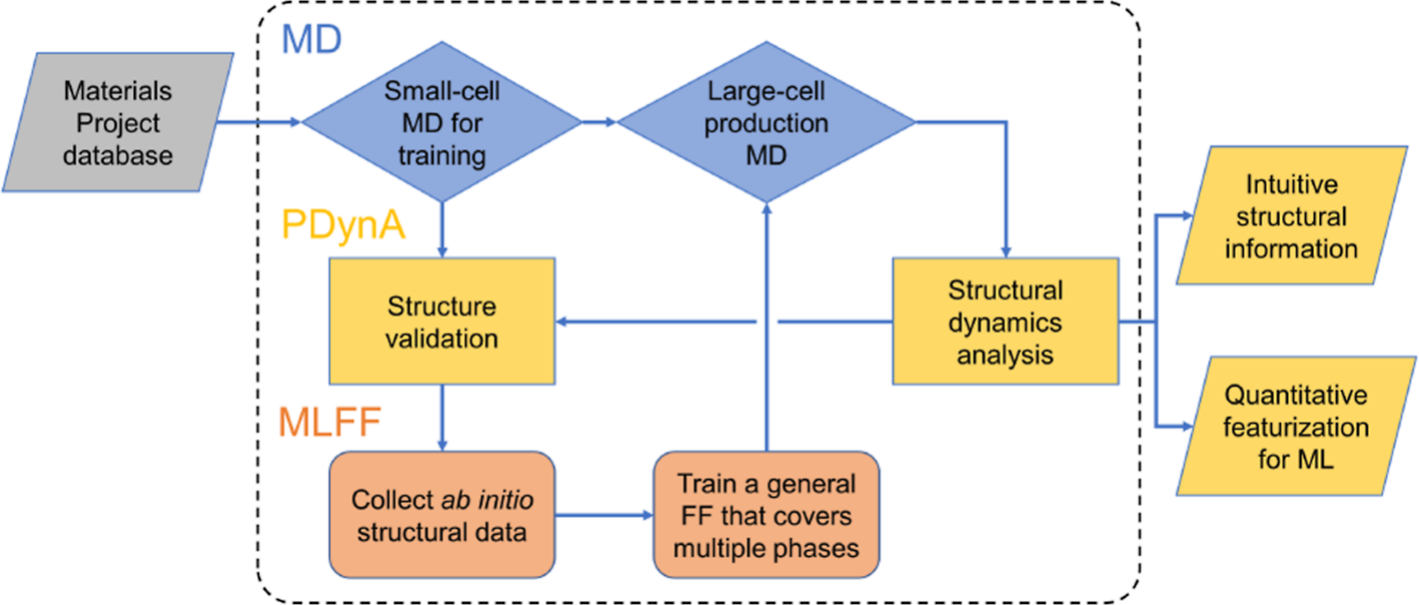
Integrated workflow used for investigating a perovskite compound of interest, combining structural analysis using PDynA of MD trajectories based on a MLFF.

All MLFF-related parameters are consistent with the defaults of VASP 6.3.0. The cutoff radius for the radial and angular representations is 5 Å, and the width of the Gaussian broadening for both representations is 0.5 Å. Validation of the general force field is achieved through another set of 6 × 6 × 6 supercell (2592 atoms) production runs at each training circumstance, and the structure is analyzed similarly to examine if the lattice parameters and global tilting patterns are consistent with the corresponding experimental measurements. Production runs are performed in a 6 × 6 × 6 supercell with the same MD parameters as the training session except that the time step is increased from 0.5 to 1 fs. The computational workflow is depicted in Figure 2.

### 2.3 MLFF Training and Trajectories for CsPbI_3_

We have demonstrated the scalability of PDynA by analyzing large-scale simulations of the inorganic perovskite CsPbI_3_. An MLFF based on the atomic cluster expansion (ACE)^43^ framework was used to simulate the material. The ACE formalism generalizes the SOAP representation used by the Gaussian approximation potentials discussed in Section 2.2. Specifically, ACE constructs a systematic body-ordered expansion of the potential energy landscape using a linear basis for symmetric polynomials and can be taken to arbitrary body order. The ACE basis is complete in principle, in contrast to SOAP or other three- or four-body representations.^44^ A detailed discussion of ACE can be found in ref (45).

The ACE model for CsPbI_3_ has been presented previously^46^ and is publicly available along with the training dataset. MD simulations of 24 × 24 × 24 supercells containing 69,120 atoms were performed using LAMMPS^47^ in the NpT ensemble. The simulation timestep was 4 fs, the system was equilibrated for 1 ns and data was then collected for 1 ns.

## 3 Results and Discussion

### 3.1 MAPbBr_3_

#### 3.1.1 Properties of the Ground State

**Figure 3:**
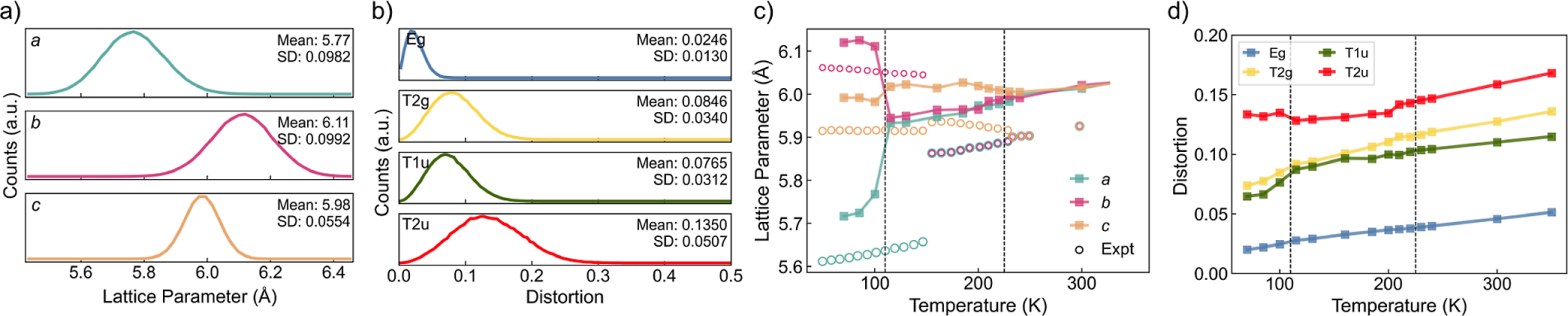
Dynamic distributions of (a) local lattice parameters and (b) octahedral distortions of MAPbBr_3_ at 100 K. Gaussian peaks are fitted to each distribution for mean and standard deviation (SD). (c) Lattice parameters *versus* temperature. Experimental values^48^ are illustrated with circles. (d) Distortion magnitudes *versus* temperature. In (c,d), the phase-transition temperatures are indicated with vertical dashed lines.

The distribution of lattice parameters and octahedral distortions of orthorhombic MAPbBr_3_ at 100 K are shown in Figure 3a,b. Both properties are evenly distributed around a mean value and can be approximated with a Gaussian distribution. Therefore, the global lattice parameters and distortion modes at constant temperature can be condensed into three and four characteristic values, respectively. The standard deviation of the lattice parameter *c* is smaller than the other two because the pseudo-cubic convention distinguishes the *c* value from *a* and *b*, by giving them different divisors. The calculated lattice parameters (Figure 3c) are approximately 0.1 Å larger than measured values.^48^

#### 3.1.2 Temperature-Induced Phase Transformations and Octahedral Tilting

The predicted α-to-β transition temperature (225 K) is well reproduced and that of β-to-γ transition (110 K) is underestimated by roughly 40 K. This determination of phase-transition temperature is achieved by performing multiple constant-temperature *NpT* MD simulations and finding the temperature range in which the tilting pattern has changed. For the octahedral distortion magnitudes, the dynamic distortion (Figure 3d) will increase with temperature due to thermal agitation. This is opposite to the distortions of the *time-averaged* crystal structure, which decrease with increasing temperature because of the higher global symmetry of the structure. The distortion magnitudes cannot uniquely distinguish between the crystallographic phases, but the information contained in the octahedral distortion has the potential for featurization covering multiple perovskite compositions, as different materials may manifest characteristic distortion patterns.

**Figure 4:**
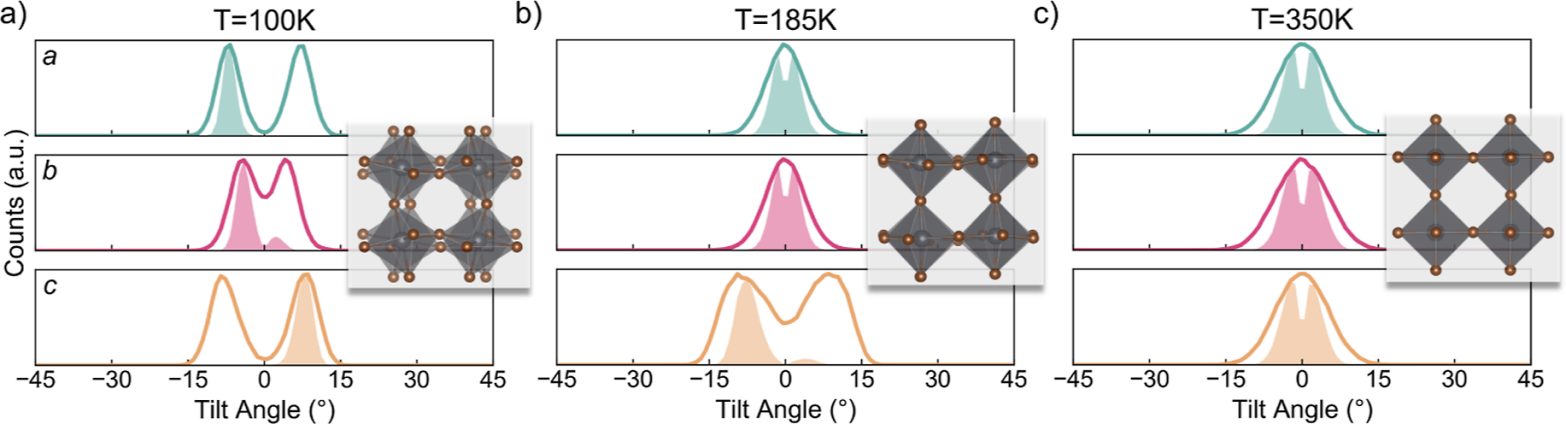
Octahedral tilting in the (a) orthorhombic phase at 100 K, (b) tetragonal phase at 185 K, and (c) cubic phase at 350 K of MAPbBr_3_. Each panel corresponds to one axis. Solid lines denote the dynamic distribution of tilting. The shaded area below the solid lines is the correlation of the tilt angle with the next nearest neighbor along the same direction, which is equivalent to the histogram of *R*_α,α_(*k*). The corresponding global Glazer tilting pattern is *a*^–^*b*^–^*c*^+^, *a*^0^*b*^0^*c*^–^, and *a*^0^*a*^0^*a*^0^, respectively.

A core functionality of PDynA is the extraction of the octahedral tilting status by isolating the distortion effect. We can directly calculate the octahedral tilt angles and the first nearest-neighbor correlation of tilting. This analysis can assign the Glazer notation^2^ of the equilibrated structure, which acts as a straightforward indicator of the crystallographic phase. For the orthorhombic phase of MAPbBr_3_ shown in Figure 4a, all three axes exhibit non-zero tilting, and an in-phase tilting pattern is observed from the *c*-axis panel (the shaded peak in the positive side is significantly larger than that of the negative side). The corresponding Glazer notation for the orthorhombic phase is *a*^+^*b*^–^*c*^–^ (*P*2_1_/*m*), and the component *a*^+^ refers to the in-phase tilting correlation in the *c*-axis. The remaining components *b*^–^*c*^–^ indicate anti-phase correlation along *a* and *b*, which have distinct octahedral tilting magnitudes. This is different from the *a*^+^*b*^–^*b*^–^ (*Pnma*) symmetry of MAPbI_3_.^49^ Thus, the tilting correlation polarity (TCP) values of this phase are approximately [−1, −1, 1].

The tetragonal phase shown in Figure 4b matches its Glazer notation *a*^0^*a*^0^*c*^–^. The octahedral tilting about *a* and *b* gives single peaks centered at 0°, and the *c*-axis has an approximate 12° anti-phase tilting with TCP values of [0, 0, −1]. Lastly, Figure 4c illustrates the tilting pattern of the equilibrated cubic phase, where all three axes possess zero-centered tilting (with a Glazer notation of *a*^0^*a*^0^*a*^0^). Moreover, all zero-centered tilting peaks (*a* and *b* axes in the tetragonal phase and all three axes in the cubic phase) have separate positive and negative correlation functions. This counter-intuitive phenomenon can be explained by the fact that even though the absolute tilting has a statistical mean of zero, the octahedra still instantaneously tilt. Nearest neighbors tilt to approximately the same angle, and this has no preferred correlation polarity (in-phase or anti-phase). Similar to the distortion and lattice parameter quantification, for each temperature, three numbers can define the global tilting pattern by performing Gaussian fitting on the absolute tilting angle distribution and another three for the computed TCP values.

**Figure 5:**
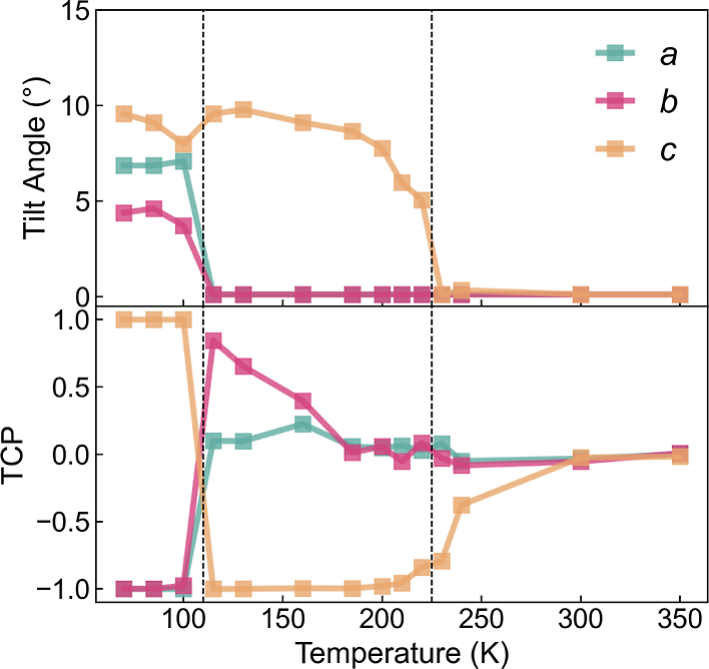
Octahedral tilt angle (upper panel) and tilting correlation polarity (lower panel) of MAPbBr_3_*versus* temperature.

The role of temperature is shown in Figure 5, with changes in the tilt angles (upper panel) and the corresponding TCP values (lower panel). All three principal axes of the cubic phase have zero-centered tilt angles, one of which becomes finite in the tetragonal phase (taking values from 5 to 10° according to the temperature), and the orthorhombic phase exhibits three distinguished non-zero tilt angles. It is also found that both phase-transition processes of MAPbBr_3_ are first-order transitions. The TCP values, however, can further reveal the spatial relations behind the octahedral tilt angles.

For the higher-temperature regime of each phase, TCP values are consistent with the formal Glazer notation. As phase-transition temperatures approached, peculiar phenomena were found. At 240 K, even though the tilt angles remain in the cubic form, the TCP value of one of the axes is driven away from zero, similar to that of the tetragonal phase. This means that the first nearest neighbors in the *c* direction tend to have anti-phase correlation rather than in-phase correlation (the Glazer plot for this temperature is shown in Figure S1a). Likewise, at 130 K close to the β–γ transition, not only the tetragonal *c*-axis but also one of the other two zero-tilting axes exhibits uneven correlation. In this case, the *b*-axis possesses an in-phase correlation with a zero-centered tilting (shown in Figure S1b). Both phenomena imply that as the cubic and tetragonal phases are cooled toward the phase transition, this dynamic tilting effect will occur and lower the effective crystal symmetry.

#### 3.1.3 Molecular Orientation: Distribution, Spatial Correlation, and Dynamics

**Figure 6:**
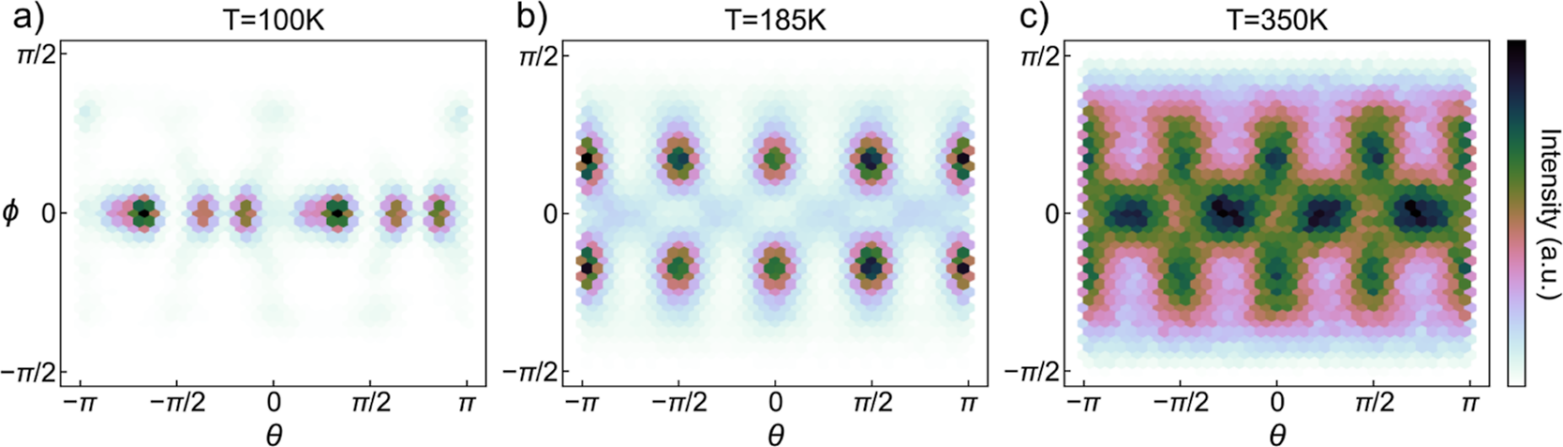
Molecular orientation distribution in spherical coordinates of the (a) orthorhombic phase at 100 K, (b) tetragonal phase at 185 K, and (c) cubic phase at 350 K of MAPbBr_3_. The orientations of the A-site molecule are projected onto the horizontal (azimuthal angle θ) and vertical (polar angle ϕ) axes.

In contrast to the strongly interconnected octahedral network, the molecular components have more orientational freedom. Each crystallographic phase has a characteristic preferred molecular orientation (MO), as illustrated in Figure 6. MA ions in the orthorhombic phase (Figure 6a) are parallel to the *ab*-plane and are confined in several local minima. A different pattern is found in the tetragonal phase (Figure 6b) where the molecules adopt eight clear preferred orientations that are symmetrical about the *ab*-plane, each forming an angle of approximately 31.4° with the *ab*-plane. The MO of the cubic phase (Figure 6c) has a much broader distribution. The MO is almost evenly spread in all directions except for those toward the nearest Pb atoms. The spherical coordinate mapping underestimates the population near the poles, especially for the cubic phase. The normalized 3D visualization is shown in Figure S2.

The molecular alignment is also affected by the adjacent molecules as quantified by the MO spatial correlation functions. Here, we will focus on the first and second nearest neighbors to reconcile with the repeating pattern of octahedra. In the orthorhombic phase, the first nearest-neighbor correlation function in the *a* direction *C*_*a*_^(1)^ (the shaded curve in panel *a* of Figure S4a) has two main populations centered at *ca.* 0.8 and −1. This means that the angle between two adjacent molecules in the *a* direction is likely to be either 37 or 180°. Interestingly, *C*_*c*_^(1)^ (the shaded curve in panel *c* of Figure S4a) has a mean value close to −1 and that of *C*_*c*_^(2)^ (the solid line) is 1, implying that the molecules are alternatively aligned in the *c* direction. The analysis is similar for the tetragonal and cubic phases. As the temperature is increased, the molecules become less correlated in space. Moreover, these correlation functions directly reflect the symmetry of the structure. In the orthorhombic phase, the molecules are correlated differently in each direction, but the symmetry relations *a* = *b* and *a* = *b* = *c* are obeyed for the tetragonal and cubic phases.

These correlation functions contain higher-dimensional information that cannot be utilized as scalar descriptors. Instead, we introduce two order parameters, namely, the alignment factor (AF) and contrast factor (CF), listed in Table 1. AF measures, for the adjacent neighbors in each direction, the difference between amounts of aligned and anti-aligned molecules. For example, AFs of the orthorhombic phase all take different values, but for the tetragonal phase in Figure S4b only AF_c_ is distinct. However, this metric does not distinguish between the tetragonal and cubic phases. Instead, the CF compares the first and second nearest-neighbor MO correlation functions. From Figure S4, CF should have the lowest value (least similar) in the orthorhombic phase and the highest value (almost identical) in the cubic phase.

**Table 1:** Characteristic Molecular Orientation (MO) Order Parameters for Each Crystallographic Phase of MAPbBr_3_, Including the Alignment Factor (AF) and Contrast Factor (CF)^a^

	MO order parameters			
phases	AF_a_	AF_b_	AF_c_	CF
[↑↑↑↑...]	1.0			1.0
[↑↓↑↓...]	–1.0			–1.0
α, 350 K	–0.05	–0.05	–0.05	0.9
β, 185 K	0.05	0.05	–0.25	0
γ, 100 K	–0.40	0.75	–1	–0.75

a Values for a 1D rod of aligned and anti-aligned molecules are shown for comparison.

**Figure 7:**
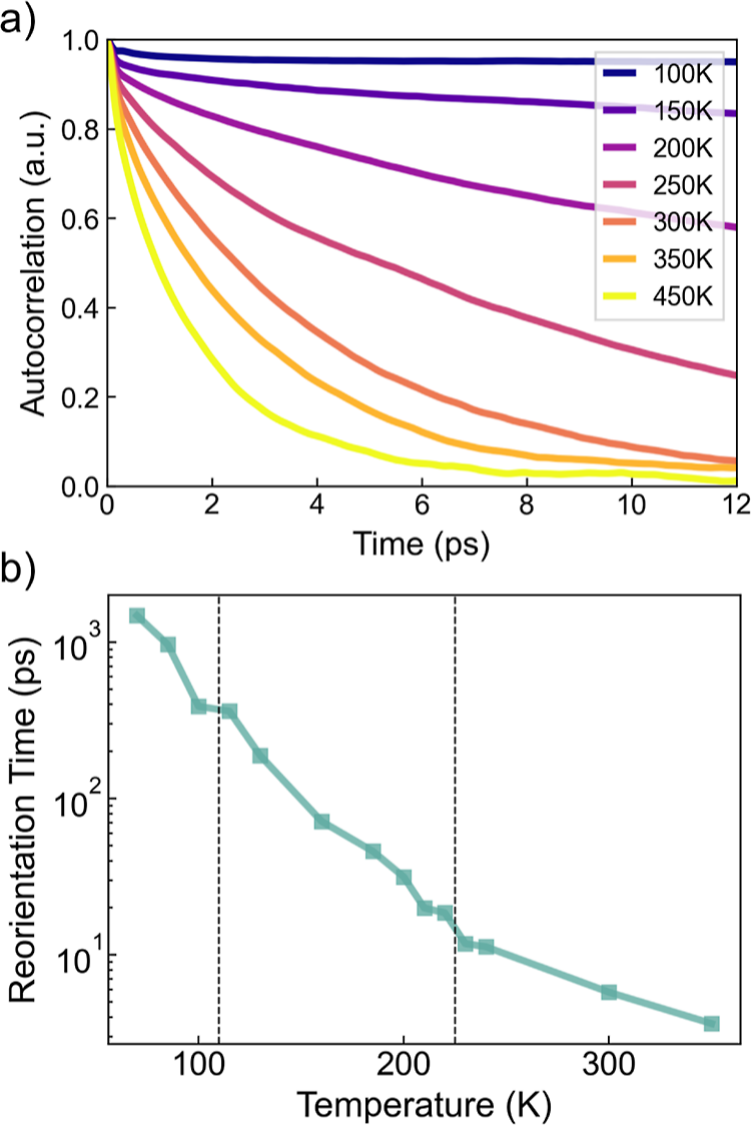
(a) Molecular autocorrelation functions versus time of MAPbBr_3_ for multiple temperatures. (b) Molecular reorientation time constants of MAPbBr_3_*versus* temperature.

The A-site molecules correlate with themselves in time as described by the autocorrelation function, Figure 7a. At lower temperatures, the molecule tends to freeze in one orientation so that the autocorrelation function stays close to 1. Higher temperatures allow larger-amplitude vibrations and hops between minima. The decay of the autocorrelation function is fitted with the exponential function with a characteristic decay time constant (see Methods). The time constants surge rapidly when the temperature is reduced and are relatively independent of the crystallographic phases. Here, we obtained molecular reorientation dynamics that are approximately an order of magnitude faster than calculations made by Jinnouchi *et al*.^28^ We attribute this to their methodology that artificially increases the H mass, which hinders the molecular rotational dynamics. The calculated room-temperature reorientation time of the MA molecule is approximately 7 ps, in good agreement with other calculations of 4–15 ps^50^ and experimental measurements of 3–14 ps.^51^

#### 3.1.4 Transient Properties

**Figure 8:**
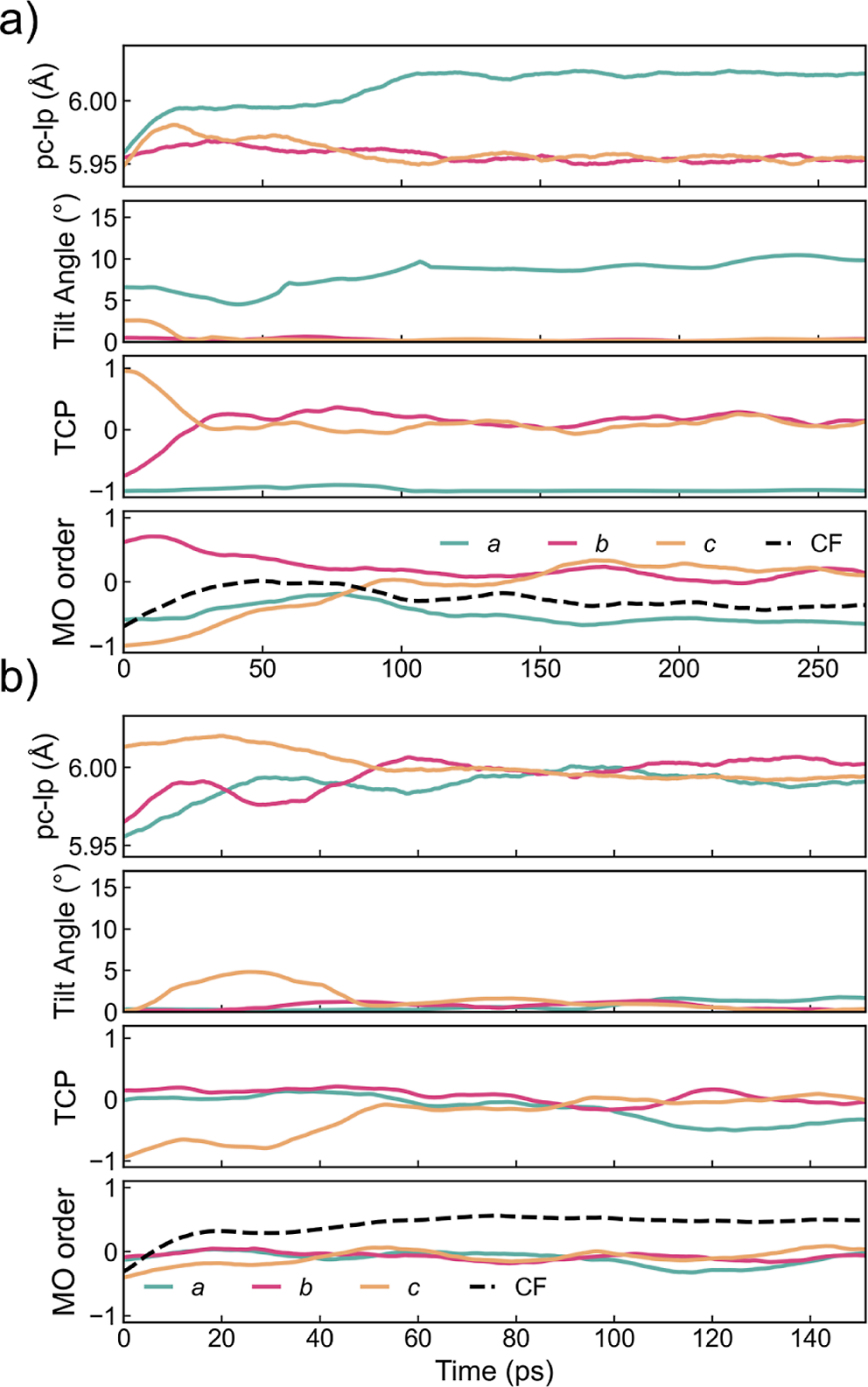
Transient structural equilibration of simulated local lattice parameters (pc-lp), octahedral tilting, tilting correlation polarity (TCP), and molecular orientation (MO) order descriptors (three AFs and a CF). (a) Orthorhombic phase equilibrated at 80 K and run at 170 K and (b) tetragonal phase equilibrated at 150 K and run at 240 K of MAPbBr_3_.

Apart from the equilibrium properties calculated from each temperature, one would also be interested in quantifying the non-equilibrium dynamics of transitions between perovskite phases. This can be achieved by introducing a temperature gradient or by changing the temperature of an equilibrated phase. We utilized the latter approach due to the slow A-site rotational dynamics. A structure was first equilibrated with constant-temperature *NpT* MD and then the atomic positions and velocities of the last frame in this calculation are used as the initial configuration of a second *NpT* MD, where the temperature is set to another temperature above the phase transition. In both orthorhombic–tetragonal and tetragonal–cubic phase transitions shown in Figure 8, a continuous evolution of all three properties can be observed, where the corresponding properties change progressively. At 170 K, the TCP values, or equivalently the pattern of coordinated tilting mode of octahedra will first converge to the tetragonal form within 50 ps, Figure 8a. Then, the octahedral tilt angles and local lattice parameters (*i.e.*, relative spacing between octahedra) will converge within 100 ps. This can be rationalized by the fact that tilting directly dictates the spacing between octahedra. Lastly, the molecules adapt their tetragonal form after 150 ps of heating. Likewise, for the tetragonal–cubic transition at 240 K (Figure 8b), all four properties transformed into their cubic form in 40 to 60 ps. Less information in this process can be extracted due to a more rapid transition, but we do observe that the reorientation of the organic A-site molecules is driven by octahedral tilting. We note that the reverse process of these two transitions is not accessible, as directly cooling the structures to a lower temperature will not trigger phase transition (the required time span for this process is longer than the allowed simulation time). This is because the kinetics of atoms at a lower temperature is slower, and the A-site molecules possess a freezing effect that impedes phase transition upon cooling.^33^

### 3.2 Analysis of Large-Scale CsPbI_3_ Trajectories

To test the capability of PDynA, we analyzed MD trajectories of CsPbI_3_ structures with a supercell size of 24 × 24 × 24 that contains 13,824 octahedra. Since the A-site of this material is inorganic, we focus on the behavior of the octahedral network.

**Figure 9:**
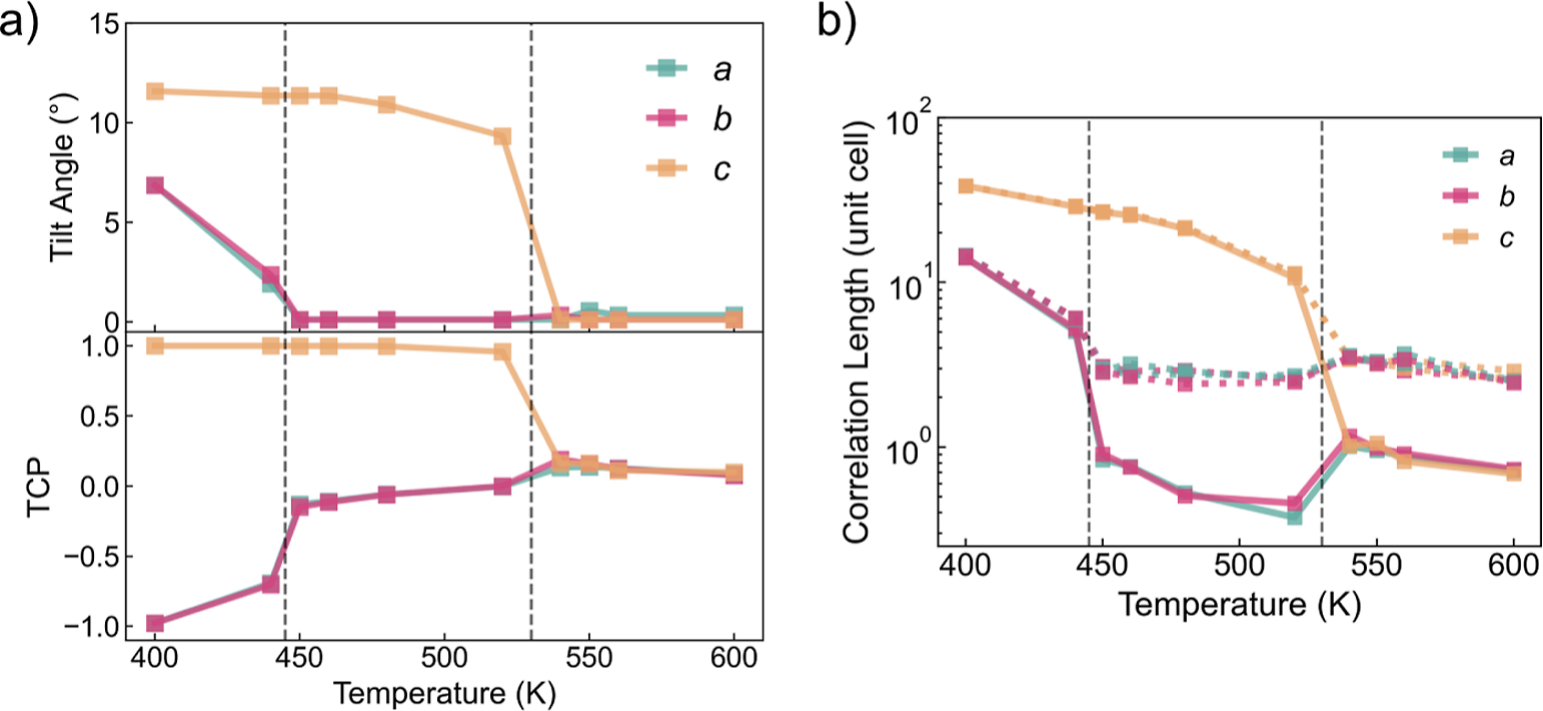
Properties of CsPbI_3_*versus* temperature, including (a) octahedral tilt angles and tilting correlation polarity (TCP) values, (b) correlation length of tilting in space, where the solid lines are diagonal terms of the spatial correlation tensor (*R*_α,β_(*k*) with α = β) and the dashed lines are the off-diagonal terms (*R*_α,β_(*k*) with α ≠ β).

The crystallographic phases found, from left (400 K) to right (600 K), are orthorhombic (*a*^+^*b*^–^*b*^–^), tetragonal (*a*^0^*a*^0^*c*^+^), and cubic (*a*^0^*a*^0^*a*^0^), Figure 9a. The corresponding tilting distributions are shown in Figure S5. Within the cubic phase, as the temperature is lowered toward the phase transition, all three TCP values increase slightly, implying that all axes exhibit subtle in-phase correlation with their neighbors. This differs from the tilting correlation behavior of MAPbBr_3_, where only one axis possesses an unequal TCP value. Local tilt domains favor in-phase correlation along all three axes of CsPbI_3_. The size of these domains can also be computed through the correlation length ξ of tilting in space, Figure 9b.

For the cubic phase, the three diagonal and six off-diagonal contributions are isotropic with respect to the principal axes. This leaves two distinct values in the correlation tensor. The off-diagonal term ξ_α,β_ is approximately 3 unit cells long, so octahedral tilting in one direction will correlate up to its third nearest neighbor in the other two directions. In contrast, the diagonal term ξ_α,α_ is mostly under 1 unit cell length, indicating the absence of correlated tilting even for the first nearest neighbor. Combining these two observations, the local tilt domain in the cubic phase has a shape analogous to a plate, which is roughly 1 unit cell thick and has a radius of 3 unit cells long. This analysis is consistent with the plate-like domains suggested from neutron scattering of CsPbBr_3_ by Lanigan-Atkins *et al.*(52) which have also been found in CH_3_NH_3_PbI_3_.^53,54^

#### 3.2.1 3D Property Maps

**Figure 10:**
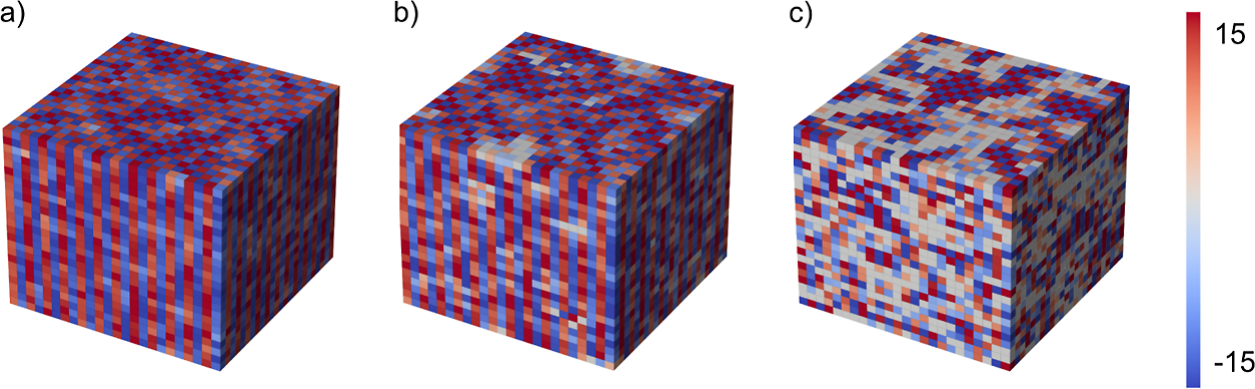
Representation of the tilt angle *c* of one frame in a dynamic trajectory. Each voxel corresponds to an octahedron in the structure of CsPbI_3_. The *c* axis is pointing up and the color bar maps between the most positive and negative tilt angles. (a) Orthorhombic phase at 400 K, (b) tetragonal phase at 480 K, and (c) cubic phase at 600 K, where on the top (001) plane, the tilt angles of neighboring octahedra in a local domain form a checkerboard (±) pattern. On the side planes, these domains are sliced to reveal their disc-like nature.

The spatial arrangement of structural features can also be plotted and analyzed directly. For this, we employ the voxel module in Matplotlib. The instantaneous octahedral tilting angles are visualized for a structural snapshot of the three CsPbI_3_ phases in Figure 10. The locked tilting of the orthorhombic phase is clearly observed in this single snapshot, consistent with the global *a*^–^*b*^–^***c***^**+**^ pattern. The tetragonal phase shows similarly locked *c* tilting, consistent with its *a*^0^*a*^0^***c***^**+**^ pattern; however, small regions of zero tilting are found. In contrast, high disorder can be seen in the cubic phase (average *a*^0^*a*^0^***a***^**0**^ pattern) with the formation of local plate-like tilting domains. These plots illustrate octahedral tilts along the *c* axis only, and correlated tilting may occur simultaneously along the other axes.

We note that the correlation length metric ξ (Figure 9b) does not take into account in-phase and anti-phase correlation effects; both modes occur with similar probability. In the tetragonal phase, a strong correlation of the *c* tilt is found in all three directions, while the *a* and *b* tilts possess weaker spatial correlations as they are in fact pure *a*^0^ axes. Lastly, the orthorhombic phase has strongly correlated tilting in all directions, consistent with the condensation of the corresponding soft phonon modes found in the high-temperature phases.

## 4 Conclusions

We have defined and implemented a set of compact structural descriptors for analyzing the average and local structures of perovskite crystals. The examples of CH_3_NH_3_PbBr_3_ and CsPbI_3_ are chosen with large MD trajectories produced using MLFFs that were trained from *ab initio* data. Importantly, the global features of each perovskite polymorph are reproduced, including structural anisotropy and octahedral tilting patterns. We gained further insights into the local correlations within the inorganic and molecular sublattices, including transient domain formation. The descriptors and systematic analysis framework are general and can be transferred to more compositionally and structurally complex perovskites.

## Supporting Information Available

A set of additional analysis plots; illustration of local lattice parameters; octahedral tilting of MAPbBr_3_ close to phase-transition temperatures; three-dimensional visualization of MO of MAPbBr_3_; fitting of the MO autocorrelation function; MO correlation function of MAPbBr_3_; and octahedral tilting of CsPbI_3_ (PDF)

## Notes

The PDynA package developed in this work is open-source and available online at https://github.com/WMD-group/PDynA (DOI: 10.5281/zenodo.7948045).
