# Supplementary material for: Structural Dynamics Descriptors for Metal Halide Perovskites
Source: J Phys Chem C Nanomater Interfaces. 2023 Aug 30;127(38):19141–51. doi: 10.1021/acs.jpcc.3c03377 (PMC10544022; doi:10.1021/acs.jpcc.3c03377)
Supplement: Supplementary file 1 — jp3c03377_si_001.pdf [file jp3c03377_si_001.pdf]

Supporting Information for "Structural Dynamics Descriptors for Metal Halide Perovskites"

Xia Liang,[1] Johan Klarbring,[1,2] William Baldwin,[3]
Zhenzhu Li,[1] Gábor Csányi,[3] and Aron Walsh[1,4,*]

[1]*Department of Materials, Imperial College London, South Kensington Campus, London SW7 2AZ, UK*

[2]*Department of Physics, Chemistry and Biology (IFM), Linköping University, SE-581 83, Linköping, Sweden*

[3]*Department of Engineering, University of Cambridge, Cambridge CB2 1PZ, UK*

[4]*Department of Physics, Ewha Womans University, Seoul 03760, Korea*

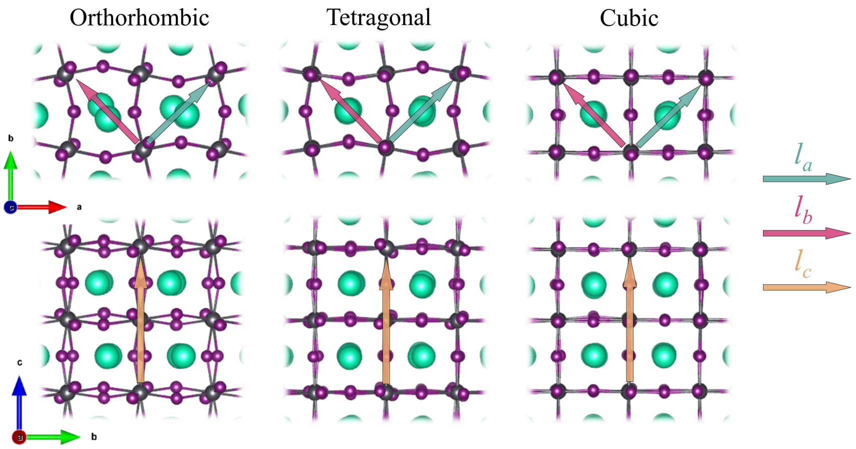

FIG. S1: Illustration of the local lattice spacing in three perovskite phases (orthorhombic, tetragonal, and cubic) defined with respect to the relative positions of the B-site cations.

* a.walsh@imperial.ac.uk

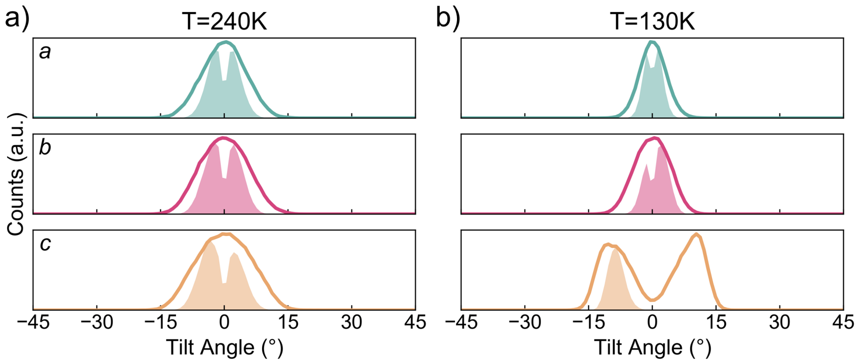

FIG. S2: Octahedral tilting at (a) 240 K and (b) 130 K in $MAPbBr_3$.

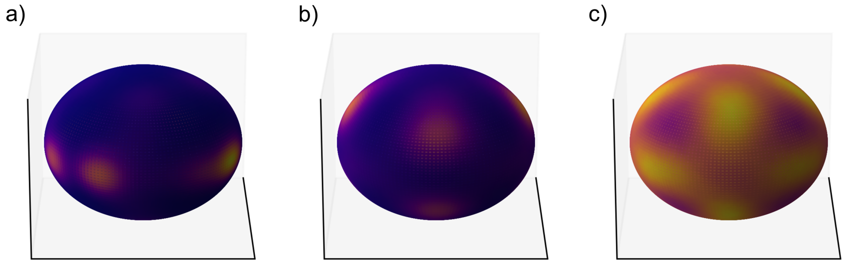

FIG. S3: Molecular orientation distribution visualised in three-dimensional space, showing the same quantity as in Fig. 6 of the main text. (a) Orthorhombic phase at 100 K, (b) tetragonal phase at 185 K, (c) cubic phase at 350 K of $MAPbBr_3$.

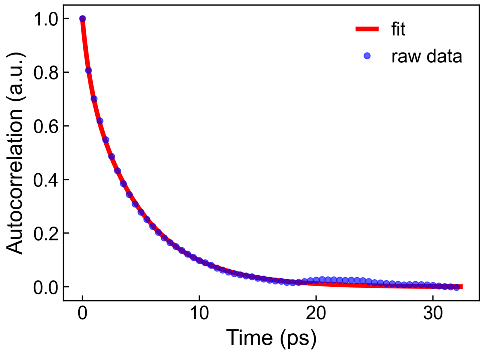

FIG. S4: Illustration of the fitting of molecular orientation autocorrelation function $A_{MO}(t)$ for CH_3NH_3.

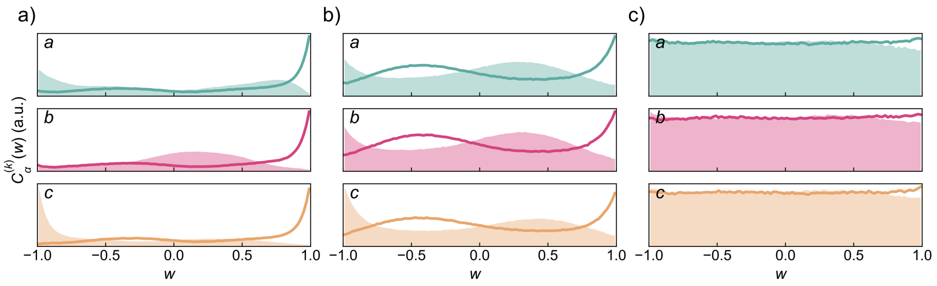

FIG. S5: Molecular orientation correlation function of $MAPbBr_3$ with (a) orthorhombic phase at 100 K, (b) tetragonal phase at 185 K, (c) cubic phase at 350 K. The shaded population in each panel refers to the first nearest neighbour correlation function $C_\alpha^{(1)}$ and the solid line is the second nearest neighbour counterpart $C_\alpha^{(2)}$.

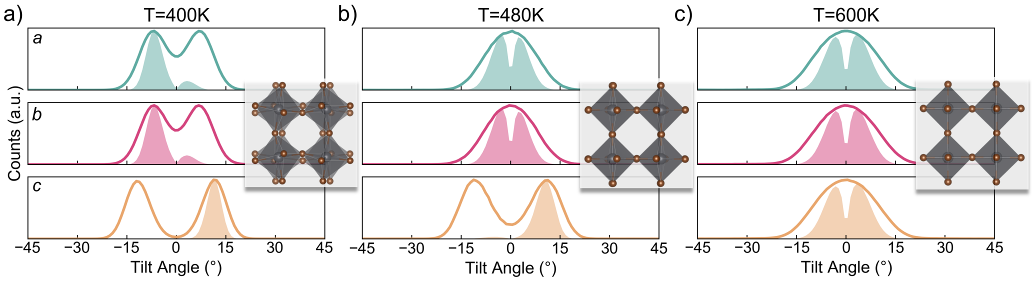

FIG. S6: Octahedral tilting in (a) orthorhombic phase at 400 K, (b) tetragonal phase at 480 K, (c) cubic phase at 600 K of $CsPbI_3$.

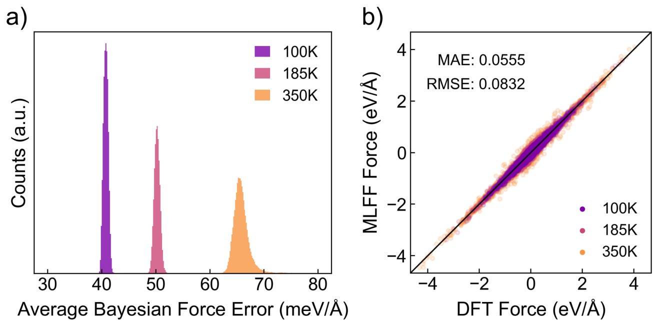

FIG. S7: (a) MLFF Bayesian error estimation of force per atom, and (b) benchmark between DFT forces and MLFF forces on each atom of $MAPbBr_3$ at 100 K, 185 K and 350 K. Three MLFF MD calculations are performed on a $4 \times 4 \times 4$ supercell at the above temperatures. Five frames are randomly selected from each trajectory. The DFT forces are calculated on these frames with the same settings as the MLFF training step, which are compared to the corresponding MLFF forces. These error distributions are normal for this type of force field.
